# Preparation of genetically engineered murine SINE RNA without endotoxin contamination

**DOI:** 10.1016/j.mex.2020.101102

**Published:** 2020-10-16

**Authors:** Xin Liu, Baixue Lv, Lifang Yan, Murad Khan, Ning Ji, Suleman Shah, Zhixue Song, Yufang Zhao, Libo Su, Xiufang Wang, Zhanjun Lv

**Affiliations:** a Department of Genetics, Hebei Key Lab of Laboratory Animal, Hebei Medical University, 361 Zhongshan East Road, Shijiazhuang 050017, Hebei Province, China; b Department of Ultrasound, Union Hospital, Tongji Medical College, Huazhong University of Science and Technology, Wuhan 430022, Hubei Province, China; c Hubei Province Key Laboratory of Molecular Imaging, Wuhan 430022, Hubei Province, China

**Keywords:** Genetically engineered RNA, Murine SINE RNA, Endotoxin, SDS-NaCl filtration method, Triton X-114 phase separation

## Abstract

RNAs have been elucidated to play the critical role in regulating gene expression and to be expected as effective drugs in the treatment of cancer and age-related diseases. RNAs are extracted by SDS-NaCl centrifugation after transformation of *E.coli* by expression vectors, which is a method to obtain genetically engineered RNAs. But the prepared RNAs by this method contain endotoxin, which limits their application *in vivo* and in cell experments. Here we improved SDS-NaCl filtration method based on SDS-NaCl centrifugation method. Endotoxin removal efficiency of SDS-NaCl filtration was nearly 4.2 times more than did SDS-NaCl centrifugation. Triton X-114 phase separation was used to reduce futher the endotoxin content of SDS-NaCI filtration-extracted RNA (from 11.25 EU/µg RNA/ml to 0.08 EU/µg RNA/ml). RNA prepared using the methods established in this paper meets the requirements for *in vivo* and cell culture experiments. Here we describe the process of preparing endotoxin-free B1as RNA from pET-B1as-DE3 *E. coli* (DE3 transformed by pET-B1as expression vector which containing a tandem SINE B1 elements) using SDS-NaCl filtration incorporating Triton X-114 phase separation.

• The endotoxin removal efficiency of SDS-NaCl filtration is higher than that of SDS-NaCl centrifugation.
• RNA prepared by SDS-NaCl filtration incorporating Triton X-114 meets the requirements for *in vivo* experiments on animals.

## Graphical abstract

**Figure:**
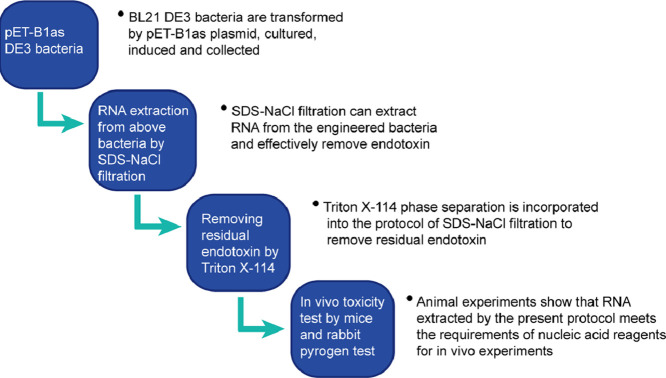

Specification table

**Table:** 

Subject Area:	Pharmacology, Toxicology and Pharmaceutical Science
More specific subject area:	*Removal endotoxin from genetically engineered RNAs*
Method name:	*Preparation of endotoxin-free genetically engineered RNAs using SDS-NaCl filtration incorporating Triton X-114*
Name and reference of original method:	*S. El-Ashram, I. Al Nasr, X. Suo, Nucleic acid protocols: Extraction and optimization, Biotechnol Rep (Amst). 12 (2016) 33–39.*
Resource availability:	*Not applicable*

## Method details

### Method summary

It is necessary to obtain genetically-engineered RNA without endotoxin contamination for animal experiments. Here, we demonstrate that the SDS-NaCl filtration method incorporating Triton X-114 can be used to obtain endotoxin-free genetically engineered RNA.

### Method description

RNAs (including microRNAs, small activating RNAs, non-coding RNAs, and more) have been elucidated to play the critical role in regulating gene expression [1], [2], [3], [4] and to be expected as effective drugs in the treatment of cancer, renal and hepatic disorders, ocular and cardiovascular disease [5,6]. Large amounts of RNA (at least mg level) are required in animal studies for the treatment of diseases. One effective way of preparing large amount of RNAs is genetically engineered RNAs prepared from *E. coli*. RNAs are extracted by SDS-NaCl centrifugation after transformation of *E.coli* by expression vectors, which is a method to obtain genetically engineered RNAs. However, RNAs prepared from *E. coli* using the above method are plagued by endotoxin contamination that, even in small quantities, can result in fever, inflammation, sepsis, tissue damage and even death *in vivo*, which limits their use *in vivo*[7], [8], [9]. Then we developed a new method referred to as ‘SDS-NaCl filtration’ which was better in aspect of endotoxin removal than that of SDS-NaCl centrifugation and resulted in a good RNA yield.

The endotoxin removal efficiency of SDS-NaCl filtration was nearly 4.2 times more than did SDS-NaCl centrifugation. The supernatant could not be completely clarified after centrifugation when using SDS-NaCl centrifugation method (11, 000 g centrifugation for 10 min). This SDS-NaCl solution has a high salt concentration (2 mol/L NaCl) and a high specific gravity, so it cannot be completely clarified during centrifugation, especially when it contains lipids [10]. The removal of insoluble substances by filtration is not affected by the specific gravity of the solution, and lipid components are easily removed by filtration because they tend to aggregate [11]. Therefore, endotoxin removal efficiency of SDS-NaCl filtration was better than that of SDS-NaCl centrifugation.

It has been proved that Triton X-114 phase separation is the effective way of removing endotoxin from recombinant proteins and plasmids [12,13]. We tested whether Triton X-114 could be incorporated into the protocol to further reduce the endotoxin content and found that Triton X-114 dramatically reduced the endotoxin content of RNA samples (from 11.25 EU/1 µg RNA/ ml to 0.08 EU/1 µg RNA/ml) extracted by SDS-NaCl filtration. Importantly, RNA extracted by SDS-NaCl filtration with Triton X-114 phase separation did not cause adverse reactions in BALB/c mice and did not induce fever when injected into rabbits. The method established in this paper can produce a large number of genetically engineered RNA without endotoxin contamination, which meets the requirements for *in vivo* experiments on animals.

Here we describe the process of preparing endotoxin-free B1as RNA from pET-B1as-DE3 bacteria (DE3 transformed by pET-B1as expression vector which contains a tandem SINE B1 elements) by SDS-NaCl filtration incorporating Triton X-114.

### Detailed protocol

#### Step 1: BL21 DE3 transformation, culture, induction, and collection

*Material*

• LB medium (containg 30 µg/ml kanamycin)
• Competent DE3 cells
• pET-B1 × 16 antisense expression vector **(**pET-B1as, containing a tandem SINE B1 elements from the mouse genome)
• Kanamycin
• LB agar plates containing 30 µg/ml kanamycin (the concentration of agar is 1.3%)
• 20 mg/ml isopropyl β- d-1-thiogalactopyranoside (IPTG) stock solution (store at −20 °C)
• 500 ml Erlenmeyer flasks
• Shaker
• High speed centrifuge
• 50 ml plastic centrifugal tubes with pointed bottom
• Spectrophotometer

#### Procedure

*Note:* All operations need to be sterile to prevent contamination of miscellaneous bacteria

1. Competent DE3 cells (0.1–0.2 ml) are transformed with 1 µl pET-B1as expression vector (0.1 µg/µl), and selected on LB agar plates containing 30 µg/ml kanamycin and cultured overnight. The bacteria transformed with pET-B1as expression vector are named pET-B1as-DE3 bacteria.
2. Choose 10–20 correct single *E.coli* bacterial colonies. Wash these colonies down with LB medium (containing 30 µg/ml kanamycin).
3. Place 200 ml LB medium (containg 30 µg/ml kanamycin) into one 500 ml Erlenmeyer flask and total need 6 Erlenmeyer flasks.
4. Divide the washed bacterial suspension evenly among 6 Erlenmeyer flasks.
5. Shake these flasks at 120 rpm at 37 °C about 3 h until the pET-B1as-DE3 bacteria are cultured to an OD _600_ of 1.0.
6. Add 2 ml IPTG stock solution into each Erlenmeyer flask and shake the flasks for another 2 h to produce B1as RNA.
7. Centrifuge the cultured bacterial suspensions at 10,000 rpm for 5 min at 4 °C in 50 ml plastic centrifugal tubes with pointed bottom.
8. After centrifugation, discard the supernatant and add the remaining cultured bacterial suspensions again until all the cultured bacterial suspensions are centrifuged. This approach typically yielded approximately 0.5 g of wet bacteria per 200 ml of cultured bacterial suspensions.

#### Step 2: RNA extraction from pET-B1as-DE3 bacteria by SDS-NaCI filtration

*Material*

• pET-B1as-DE3 bacteria (*E. coli* DE3 transformed by pET-B1as expression vector)
• 1.5% SDS solution (10 mM Tris-10 mM Nacl-1.5% SDS, Ph7.4)
• Diethy pyrocarbonate (DEPC)
• Absolute ethyl alcohol and 75% ethanol
• RNase inhibitor (40 U/µl)
• RNase-free DNase I kit (including 10 × DNase I buffer, RNase-free DNase I (1 U/µl))
• Slow filter paper (10–15 µm)
• Double-distilled H2O (DDW)
• 50 mg/ml lysozyme (store at −20 °C)
• Normal saline (0.9% NaCl)
• 4 mol/L NaCl
• High speed centrifuge
• 50 ml plastic centrifugal tubes with pointed bottom
• Spectrophotometer

*Procedure*

1. After the bacterial suspensions are centrifuged, the pellets are made into 50% bacterial suspension (in volume) using normal saline. The specific method is as follow: the pellets in the six 50 ml plastic centrifugal tubes are suspended with 2 ml normal saline, determine the volume, and then add the appropriate amount of normal saline to make the final concentration of wet bacteria is 50%.
2. Divide 1 ml 50% bacterial suspension into one 50 ml plastic centrifugal tube, add 20 µl 50 mg/ml lysozyme to one tube and place the tubes on ice for 5 min to get the lysed bacteria.
3. Add 9 ml 1.5% SDS solution (final concentration about 0.7%) to the lysed bacteria.
4. Add 6 µl DEPC to prevent RNA degradation at room temperature.
5. Add 10 ml 4 mol/L NaCl to each tube and mix it.
6. Use slow filter paper to filter the bacterial lysates at 4 °C.
7. Use three volumes of absolute ethyl alcohol to precipitate the filtered solution for 30 min at room temperature and then centrifuge for 10 min at 10,000 rpm.
  *Note: Precipitating the filtered solution process should not be carried out at 4* °*C, otherwise a large amount of salt precipitation will occur, interfering with the further purification process*.
8. Use 75% ethanol to wash the precipitate twice.
9. Use hot air from a hair dryer to dry the RNA.
  *Note: The drying process cannot be performed via natural evaporation because of each tube having a large amount of precipitation and the long time being required in natural evaporation drying. After the ethanol volatilization during the natural drying process, the water-soluble RNA state will be formed, leading to RNA degradation.*
10. Prepare 2 ml 0.5 × DNase I digestive solution: DDW 1.775 ml, 0.1 ml 10 × DNase I buffer, 0.1 ml RNase-free DNase I (final concentration 0.05 U/ml), 25 µl RNase inhibitor (final concentration 0.5 U/ml)
11. Add the above 2 ml 0.5 × DNase I digestive solution into the dried RNA, mix, keep for 30 min at 37 °C, collect the supernatant after centrifugation (10,000 rpm 10 min).
12. In addition to RNA, the supernatant (DNase I digested supernatant) contains degraded DNA and residual proteins. All DNase I digested supernatant obtained is treated with SDS hot-phenol method [10] to remove residual proteins and degraded DNA if without further purification by Triton X-114.
  *Note:* If further Triton X114 removal of endotoxin is required, the following steps are performed: take out 0.1 ml of the supernatant, use SDS hot-phenol method [10] to remove residual proteins, centrifuge at 10,000 rpm for 10 min to get a SDS-phenol supernatant, use three volumes of absolute ethyl alcohol to precipitate the SDS-phenol supernatant for 30 min at 4 °C, centrifuge for 10 min at 10,000 rpm, wash the precipitate twice using 75% ethanol (remove phenol, SDS and degraded DNAs). Dry the precipitate using hot air from a hair dryer, add the DDW to recover the volume of SDS-phenol supernatant, determine the OD values at 260 nm and 280 nm, calculate the ratio of OD260/OD280 and concentration of the prepared RNA. The remaining DNase I digested supernatant is prepared into 1 mg/ml with DNase I buffer (0.5 ×) according to RNA concentration determined by above to get 1 mg/ml RNA solution.

#### Step 3: removing residual endotoxin by Triton X-114 phase separation

*Material*

• Triton X-114
• 3 mol/L sodium acetate (pH 6.0)
• Absolute ethyl alcohol and 75% ethanol
• 10% SDS
• Water-saturated phenol
• Chloroform
• Thermostatic metal bath
• Shaker
• High speed freezing centrifuge

#### Procedure

1. Add one-tenth volume of 3 mol/L sodium acetate (pH 6.0) into the above 1 mg/ml RNA solution.
2. Add one-thirtieth volume of Triton X-114 and mix.
3. Incubate and shake the mixture for 20 min at room temperature.
4. Move to a metal bath at 50 °C for 10 min.
5. Keep at room temperature for 5 min.
6. Centrifuge the mixture at 10,000 rpm 10 min at 23 °C using high speed freezing centrifuge.
7. Collect the supernatant, remove the residual proteins using SDS hot-phenol methods [10]. Briefly, add one-tenth volume of 10% SDS, add a half volume of water-saturated phenol, keep 30 min at 60 °C, after cooling add one quarter volume of chloroform, mix the mixture by inverting, centrifuge the mixture at 10,000 rpm 10 min.
8. Collect the supernatant, precipitate the supernatant with three volumes of absolute ethyl alcohol for 30 min at 4 °C
9. Centrifuge for 10 min at 10,000 rpm and wash the precipitate twice using 75% ethanol.
10. Dry the precipitate using hot air from a hair dryer to get endotoxin-free RNA.

## Method validation

The novel combination of SDS-NaCl filtration and Triton X-114 phase separation can extract engineered RNA from *E.coil* transformed by expression vectors and effectively remove endotoxin at the same time. SDS-NaCl filtration was performed to extract RNA from pET-B1as-DE3 bacteria and endotoxin content of RNA was 11.25 EU/1 µg RNA/ml. The OD260 / OD280 ratio of the RNAs extracted by SDS-NaCl filtration was approximately 2.0, which suggests that the method is capable of producing RNA with sufficient purity and implies that contamination by DNA and protein can be ignored.

SDS-NaCl filtration is the effective way of removing endotoxin. However, RNA extracted by SDS-NaCl filtration still contains some endotoxin (11.25 EU/ 1 µg RNA/ml). We next sought to test whether Triton X-114 could be incorporated into the SDS-NaCl filtration protocol to remove residual endotoxin and found that Triton X-114 dramatically reduced the endotoxin content of RNA samples (from 11.25 EU/1 µg RNA/ ml to 0.08 EU/1 µg RNA/ml) extracted by SDS-NaCl filtration.

RNA extracted by SDS-NaCl filtration with Triton X-114 phase separation was injected intravenously into three New Zealand rabbits (50 µg RNA/kg). The total increased body temperature of three rabbits was 0.09 °C. Based on the injected dose of RNA, this increase suggests that the endotoxin content of the RNA was lower than 1 EU/50 µg RNA. BALB/c mice injected intravenously with RNA extracted by SDS-NaCI filtration with Triton X-114 had no any adverse effect. Therefore, this new protocol-extraction of genetically engineered RNA by SDS-NaCl filtration combined with Triton X-114-meets the requirements for *in vivo* experiments.

**Fig. 1:**
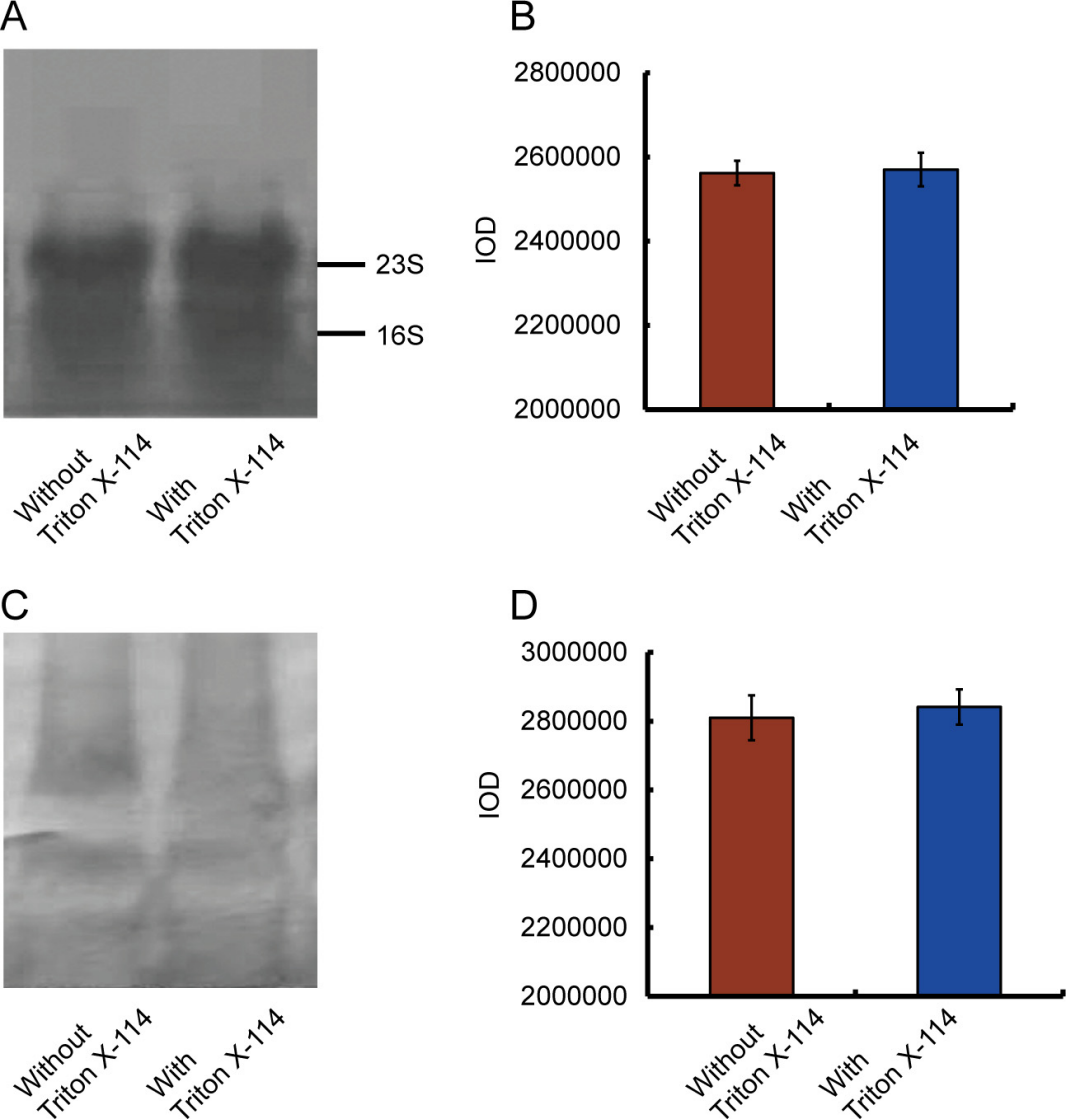
Effects of Triton X-114 treatment on RNA integrity and B1as RNA yield. (**A**) Representative methylene blue stain of total RNAs without or with Triton X-114 treatment. The ratio of 23S to 16S without (lane 1) and with Triton X-114 (lane 2) treatment were approximately 2:1, indicating that Triton X-114 treatment did not cause RNA degradation. (**B**) Quantification of RNA (IOD value) per lane in Fig. 1A (means of three independent experiments). (**C**) Representative result of northern blotting of B1as RNA without (lane 1) or with Triton X-114 (lane 2) treatment. (**D**) Quantification of RNA (IOD value) per lane in Fig. 1C (means of three independent experiments). (For interpretation of the references to color in this figure legend, the reader is referred to the web version of this article.)

The effects of Triton X-114 treatment on RNA integrity and B1as RNA yield were examined using northern blotting. Total RNA was treated without or with Triton X-114, electrophoresed and transferred into nylon membrane. The ratio of 23S to 16S without (Fig. 1A, lane 1) and with Triton X-114 treatment (Fig. 1A, lane 2) were approximately 2:1, indicating that Triton X-114 treatment did not cause RNA degradation. The comparison before and after Triton X-114 treatment proved that the recovery rate of B1as RNA is approximately 70–80%.

## Declaration of Competing Interest

The authors declare that they have no conflicts of interest related to this work.
